# Functional and Objective Audiovestibular Evaluation of Children With Apparent Semicircular Canal Dehiscence–A Case Series in a Pediatric Vestibular Center

**DOI:** 10.3389/fneur.2019.00306

**Published:** 2019-04-02

**Authors:** Soumit Dasgupta, Sudhira Asanka Bandara Ratnayake

**Affiliations:** Department of Paediatric Audiology and Audiovestibular Medicine, Alder Hey Children's NHS Foundation Trust, Liverpool, United Kingdom

**Keywords:** semicircular canal dehiscence, third window, audiovestibular, video head impulse, pediatric, high resolution CT

## Abstract

Semicircular canal dehiscence is a bony abnormality in the otic capsule especially involving the superior semicircular canal. Since its identification in 1998, there is significant research regarding the pathology in the adult population. This condition generates a third window effect that is well–described in the literature. However, the entity is rare in the pediatric population with limited research. Difficulties encountered in children are obtaining a direct history that is essential for the diagnosis followed by neurovestibular tests that may be difficult to perform. This study presents observations regarding different clinical and diagnostic aspects of semicircular canal dehiscences in children as a retrospective audit in a tertiary pediatric vestibular center. Of 580 children assessed in a 30 months period undergoing comprehensive functional and objective audiovestibular assessment, 13 children (2.2%) were detected to possess radiological semicircular canal dehiscences (high resolution computed tomography scans at 0.625 mm slices reformatted in the axial, coronal and sagittal planes). The right superior semicircular canal was most commonly affected (66.6%). There were 4 bilateral semicircular canal dehiscences. Clinical suspicion of the condition was raised with reliable surrogate history from carers or from older children (100%), a mixed or conductive hearing loss (80% of hearing losses) in the presence of normal impedance audiometry (92.3%), normal transient otoacoustic emissions (84.6%) on the side of the dehiscence and the presence of replicable pathological saccades in the video head impulse test (76.9%). Disequilibrium symptoms and typical third window symptoms were absent or difficult to elicit in children (46.15 and 30.76% respectively). Only 3 (0.5%) fulfilled the adult criteria of a superior semicircular canal dehiscence syndrome. The abnormal video head impulse test characterized by pathological saccades may affect other non-dehisced ipsilateral canals. Semicircular canal dehiscences are rare in children but may be considered as an etiology for hearing losses and imbalance. Children with semicircular canal dehiscence may present differently from the classical superior semicircular canal dehiscence syndrome found in adults.

## Introduction

The pathological entity of superior semicircular canal dehiscence (SSCD) since its first description by Minor (1) has seen immense interest and research. Originally described in patients with sound or pressure induced vertigo and nystagmus (Tullio's and Hennebert's phenomenon) with symptoms of chronic disequilibrium, the clinical features that subsequently came into light included oscillopsia, auditory features including conductive hearing loss, autophony, conductive dysacusis including gaze evoked tinnitus, pulsatile tinnitus, low frequency hearing loss, phonophobia, and aural fullness (2). Vestibular symptoms may be absent (3).

The pathophysiology of the auditory features of SSCD can be attributed to the pathological presence of a third window in addition to the natural two windows for maintaining integrity of inner ear sound conduction. The pathological third window shunts away a proportion of the sound energy delivered at the stapes footplate-oval window interface and thus from the cochlea resulting in abnormally elevated air conduction thresholds in pure tone audiometry. The same mobile third window lowers the impedance or pressure difference of the cochlear traveling wave between the scala vestibuli and the scala tympani in the inner ear by allowing a new path for the sound to enter the inner ear thereby generating enhanced bone conduction thresholds in bone conducted pure tone audiometry (4). The vestibular features in SSCD are due to its enhanced vulnerability to pressure changes created by sound conduction. Other conditions in the otic capsule generating a similar third window effect are posterior and lateral semicircular canal dehiscences, enlarged vestibular aqueducts, the X linked gusher syndrome, facial nerve canal dehiscences, dilated bony internal auditory meatus, and dehiscent carotid canals.

The etiology of SSCD appears to be developmental and congenital (5) and it has been suggested that thinning or dehiscence of the bone in the otic capsule over the semicircular canal is more prevalent in children younger than 12 months and with age, the semicircular canals may develop more bony covering (6). Carey et al. suggested that there may be a malformation in neonatal bony development (7). Head trauma may also lead to a dehiscent semicircular canal (5). Congenital SSCD may be accompanied with deficient tegmens and dysplasia found in some syndromic conditions (5). Nadgir et al. (8) however, proposed a predominantly acquired etiology and suggested that frank dehiscences increased with age due to bony demineralization. The debate is unresolved.

The diagnosis of the condition in addition to the clinical features rested primarily on imaging with a high resolution CT scan. However, a CT scan is not sufficient on its own to confirm a diagnosis as many as 9% may have a dehiscence on a coronal temporal bone CT with a 1 mm slice, many of whom are asymptomatic (5, 9) and unless reformatted with slices <0.625 mm in the Poschl and Stenver's views, CT scan may still over diagnose the condition (5). Thus, it is crucial to consider physical symptoms and physiological evidence of a third window (5).

Whilst auditory symptoms suggest a third window phenomenon, subsequent research has shown that vestibular evoked myogenic potentials (VEMPs) are reliable, sensitive and specific indicators of a pathological third window due to lowering of impedance in the vestibular system as regards sound and pressure resulting in lower thresholds and higher amplitudes in the VEMPs (2). The cervical VEMP was the first VEMP identified and now it has been discovered that air conducted ocular VEMPs have a sensitivity and specificity of more than 90% to detect a pathological third window (10). The use of VEMPS in semicircular canal dehiscences in children has limited evidence.

SSCD is very well-described in the adult population, however, evidence of the condition in the pediatric population is evolving. Data regarding quantification of vestibular function in the condition in children is meager. Until 2007, only 3 children were described with the condition (3). Chen et al. (11) in a retrospective cohort analysis in the pediatric population analyzed 131 temporal bones in children presenting with hearing loss over the age of 3 years and observed that 18 (15%) showed CT evidence of semicircular canal dehiscence with 13 SSCD and 5 posterior semicircular canal dehiscences (PSCD). The series did not include vestibular function test data. Lee et al. (12) in a cohort of 7 children observed that hearing loss was the predominant presenting feature with disequilibrium as the next common presenting complaint in children. Third window features could only be elicited in the 3 oldest children. Of vestibular function tests, VEMPS were performed which were found to be abnormal in the majority. Meiklejohn et al. (13) studied live and cadaveric temporal bone specimens in children younger than 7 years and observed that the prevalence of radiographic semicircular canal dehiscence declined with increased age in children, reinforcing the idea that the otic capsule thickens with age. Normal, mixed and sensorineural hearing losses (SNHL) were reported in the live cohort of 19 children, however, there were no children with third window features or with disequilibrium. Sugihara et al. (14) identified an incidence of 6.2% of SCDS in the pediatric population in a large multicenter review. This study very interestingly did not suggest that other inner ear anomalies are significantly associated with SCDS in children. Hagiwara et al. (15) pointed out that radiographic evidence of semicircular canal dehiscence does not necessarily suggest a semicircular canal dehiscence syndrome i.e., clinical symptoms. There have been some isolated case reports also (3, 16, 17).

The present study was a retrospective study that looked into audiovestibular quantification of semicircular canal dehiscences in the pediatric population in detail for the first time in a tertiary pediatric balance center in the United Kingdom to analyse the patterns of clinical presentation, auditory and vestibular functions and the relationship between behavioral audiometry with objective vestibular function.

## Patients and Methods

### Patients

A retrospective case note audit was performed in children who underwent vestibular quantification in the pediatric age group as a service improvement exercise in Alder Hey Children's NHS Foundation Trust, a tertiary children's hospital in Liverpool, United Kingdom between the period of February, 2016 and July, 2018. These children attended the secondary and tertiary audiovestibular clinics in the hospital. The study was approved by the clinical audit department of the hospital. Being a retrospective audit, the study population did not require ethical committee approval. Only children above the age of 5 years up to 17 years were included in the case series. Dysplastic vestibular pathologies with hypoplasia/aplasia of canals with dehiscences as shown in imaging were excluded.

### Methods

#### Anamnesis

History for audiovestibular manifestations in young children are often surrogate and obtained from carers. This was attempted to be as comprehensive as possible with accounts from both carers, from schools as well as children themselves wherever obtainable. A subjective narrative is extremely important in children with balance problems or hearing losses. A frank history of disequilibrium or disorientation that adults can generally narrate so well is frequently impossible from children including teenagers. Therefore, since balance in children can be observed by others and balance problems may lead to predictable behavior, an accurate description from carers were absolutely crucial which in turn can be fairly reliable indicators of the problems. Similarly with hearing loss, a deficit in communication and educational performance were deemed as a key indicator of a positive history. Some children narrated symptoms of conductive dysacusis. Key points in history are shown in Table 1.

**Table 1:** Symptoms of pediatric vestibular disease.

• Obvious dizziness/vertigo/lightheadedness (usually describable by children above 8 years of age) • Fright or pallor • Clutching at objects to steady oneself • Bumping into things • Clumsiness • Sudden very brief lasting falls with immediate complete recovery • Periodic episodes of nausea or vomiting ± migrainous features • Delayed motor functions • Loss of postural control • Difficulty with ambulating in the dark • Difficulty with or avoidance to ride a bike or in amusement park rides due to imbalance • Abnormal movements during walking, running • Abnormal behavior observed up by significant others (care giver, school or peer group) • Difficulties in challenging movements (swimming, dancing) • Oscillopsia • Difficulties in challenging visual environments for example in superstores and in crowded places • Poor head eye or hand eye coordination • Third window symptoms if described by older children—conductive dysacusis (for example hearing one's own footsteps), gaze evoked tinnitus (audible eye movements), autophony (altered perception or perverted self-monitoring of own voice), Tullio's phenomenon (dizziness on hearing loud sounds), Hennebert's phenomenon (pressure induced dizziness for example on coughing and sneezing), pulsatile tinnitus (tinnitus that is synchronous with pulse beat)

#### Audiovestibular Quantification

With full verbal informed consent, all children underwent otoscopy, tympanometry, stapedial reflex tests, pure tone audiometry; transient otoacoustic emissions (OAE) and a neurovestibular examination that included observation of the subjective visual vertical (measurement of head tilt with respect to earth's vertical to assess static gravitational sensor function); a full videonystagmography (VNG) examination with and without visual fixation to observe and measure smooth pursuits and saccades (to assess central vestibular function), eccentric eye movements (to assess nystagmus); the passive head shake (appearance of post headshake nystagmus) and the head heave test (translational counter part of the head thrust test for utricular function); the amplitude and symmetry of ocular counter rolling (ocular movements in response to head roll to assess gravitational sensor function). All these tests were video recorded. Further tests performed were the vHIT in all 6 semicircular planes; the VOR suppression test and office rotatory chair tests under VNG control and the vestibulo-spinal test battery including the Romberg, the Unterberger, the tandem gait and the one legged stance as well as sharpened Romberg's test with and without proprioception to eliminate as much proprioception as possible (i.e., in a foam cushion) removed. Some children also underwent the mastoid vibration test under VNG control to yield additional information on low frequency lateral semicircular canal responses and some older children were subjected to the Dix Hallpike, the supine roll test and the deep head hanging test to test for benign positional paroxysmal vertigo (BPPV) especially when they complained of positional dizziness. Caloric testing was not performed due to its logistic issues when performed in children especially taking into account the distress caused in children by caloric testing. At the time of the study, the center did not possess VEMPS that it now does. One of the reasons as to why the case review was undertaken was to assess whether the service provisions available at that time permitted the diagnosis of superior semicircular canal dehiscences in children that in turn would suggest areas for improvement.

In addition, a full neurological examination was performed in all children as well as a full oculomotor and musculoskeletal examination as part of the holistic test battery. The history and the neurotological investigations were performed by the authors who are experienced clinicians specializing in pediatric vestibular disorders who peer reviewed each other's observations to reach a consensus. The audiovestibular examination and tests are given in Table 2 which also qualifies the tests.

**Table 2:** The pediatric audiovestibular test battery.

**Audiological tests**	**Vestibular tests**
• Pure tone audiometry with masking • Tympanometry • Stapedial reflexes • Otoscopy • Transient otoacoustic emissions	• Full neurological examination • Musculoskeletal examination • Full oculomotor examination • Assessment of subjective visual vertical • Videonystagmography with and without visual fixation for head shake, head heave, ocular counter rolling and ectopic eye movements • Video head impulse test • Vestibulo-spinal test battery with and without foam cushion for Romberg, Unterberger, tandem gait; one legged stance and sharpened Romberg • Office rotatory chair tests and suppression of visual fixation • Mastoid vibration test • Dix Hallpike, supine roll and deep head hanging tests

*VNG software was used to video record and play back for head shake, head heave, ocular counter rolling, mastoid vibration, positional tests and suppression of visual fixation tests; Unterberger test: child is asked to walk/jog in place with eyes closed; Sharpened Romberg's test: placing one heel in front of toe as in tandem gait but not moving with eyes open and closed with and without proprioception*.

Pure tone audiometry involved measurement with air conduction and bone conduction with the acoustic stimuli delivered through TDH39 head phones and thresholds up to 20 dBHL were considered as normal. Pure tone air conduction thresholds were performed from 500 Hz up to 6k Hz for every child to obtain as much information on hearing as possible especially because meaningful speech containing consonants are mainly between 4 and 6 kHz. For this paper, pure tone averages between 500 Hz and 4 kHz in every child was averaged for air and bone conduction thresholds. Bone conduction thresholds were performed wherever indicated especially with masking but occasionally were difficult to obtain in young children due to intolerance or the complexity of the task. Transient otoacoustic emissions were measured at stimulus intensity of 80–88 dBSPL on both ears by equipment from Otodynamics; VNG and vHIT were performed by the ICS Impulse software from Otometrics. For the vHIT, an abnormal result was denoted by lower than normal vestibulo-ocular reflex gain (VOR) in the semicircular canals with catch up overt and covert saccades and by normal VOR gain with replicable and repeatable overt and covert saccades. Normal VOR gain with catch up refixation saccades after vestibular pathology have been recently identified as an important observation in the evolving literature with the vHIT (18). A minimum of 10 thrusts in different semicircular canals (laterals and right anterior left posterior RALP and left anterior right posterior LARP) were achieved to draw meaningful conclusions. The normal gain in the lateral semi-circular canal was considered to be 0.8 to 1 and that of the vertical canals to be 0.7 to 1. It is worth pointing out that in a new and emerging evidence in the pediatric population, the vertical canal gains involving RALP and LARP may be lower than in the adult population (19) that could be to contamination by a developing cervical neck musculature. All children presenting with hearing loss underwent the full set of aetiological investigations as recommended by the British Association of Audiovestibular Physicians (20) that included MRI and genetic testing, ophthalmological examination, drawing of family tree and blood investigations, looking into metabolic conditions that can cause hearing loss and autoimmune ear disorders. These were unremarkable in our case series diagnosed with canal dehiscence.

#### High Resolution CT

Based on anamnesis and audiovestibular information; children with third window symptoms, hearing losses and balance problems either alone or in combination with and without tinnitus were subjected to a high resolution CT scan to obtain a comprehensive idea about the bony otic capsule. The slices were 0.625 mm thick with a high spatial filter. Axial, coronal and sagittal reconstructions were performed. The investigation was performed by a senior radiology consultant colleague specializing in pediatric head and neck radiology.

#### Statistical Methods

Following the diagnostic algorithm, children with CT evidence of semicircular canal dehiscences were subjected to descriptive analysis performed by Quick Statistics Calculators, an online digital portal (https://www.socscistatistics.com/tests/).

## Results

**Figure 1:**
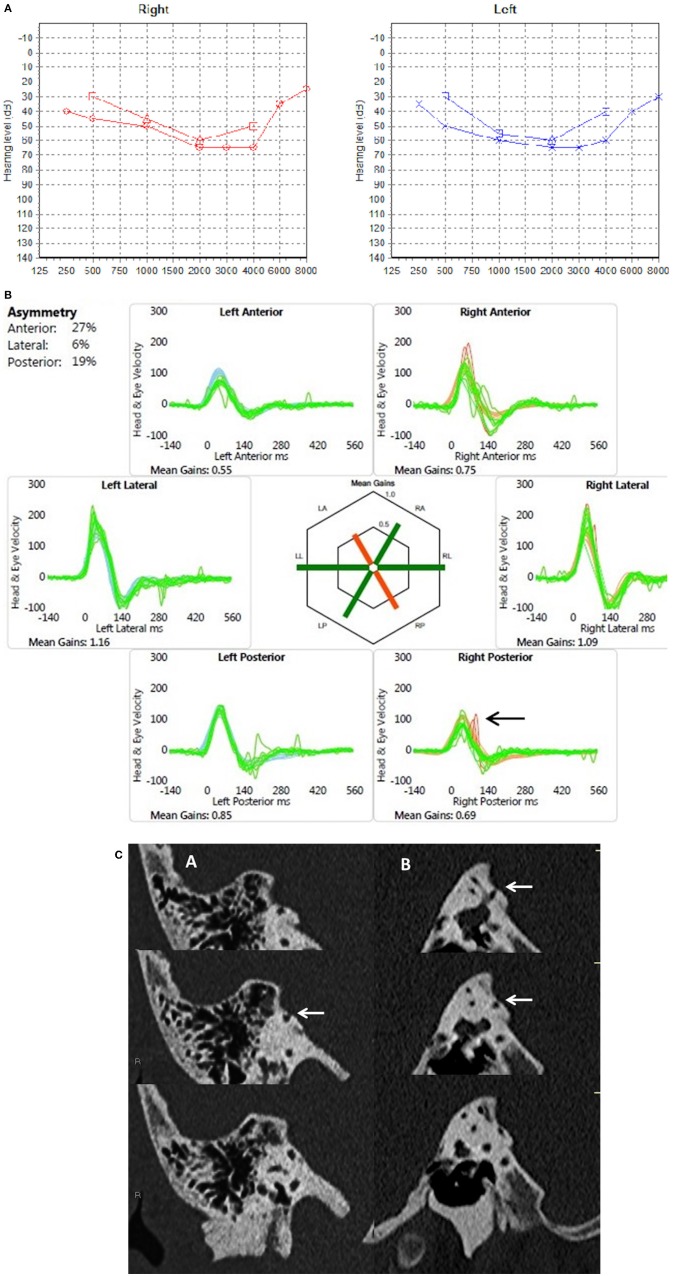
**(A)** PTA in a child presenting with mixed hearing loss (child 1). **(B)** Video head impulse test in same child with at least 2 covert replicable saccades in the right posterior semicircular canal (arrow). **(C)** Coronal (A) and sagittal (B) reconstructions of a high resolution CT scan with consecutive slices demonstrating dehiscence of the right posterior semicircular canal (arrows).

**Figure 2:**
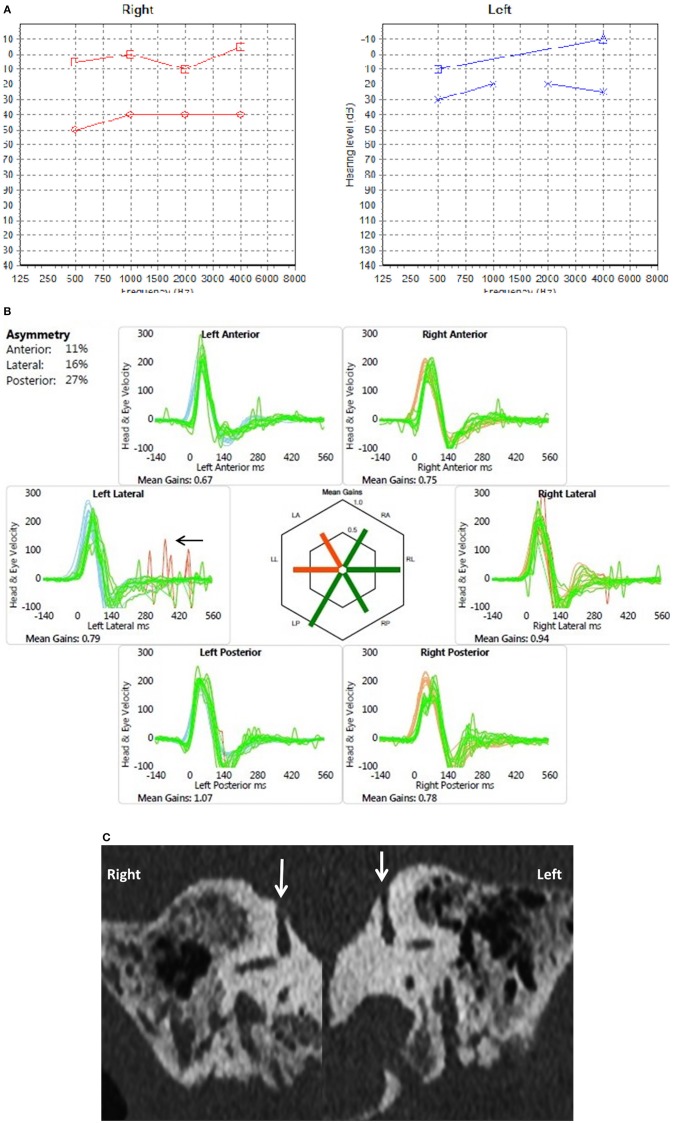
**(A)** PTA in a child presenting with conductive hearing loss on both sides (child 6). **(B)** Video head impulse test in the same child with overt replicable saccades in the left lateral semicircular plane (arrow). **(C)** Coronal reconstruction of High resolution CT scan shows dehiscence of the superior semicircular canal on both sides (arrows).

**Figure 3:**
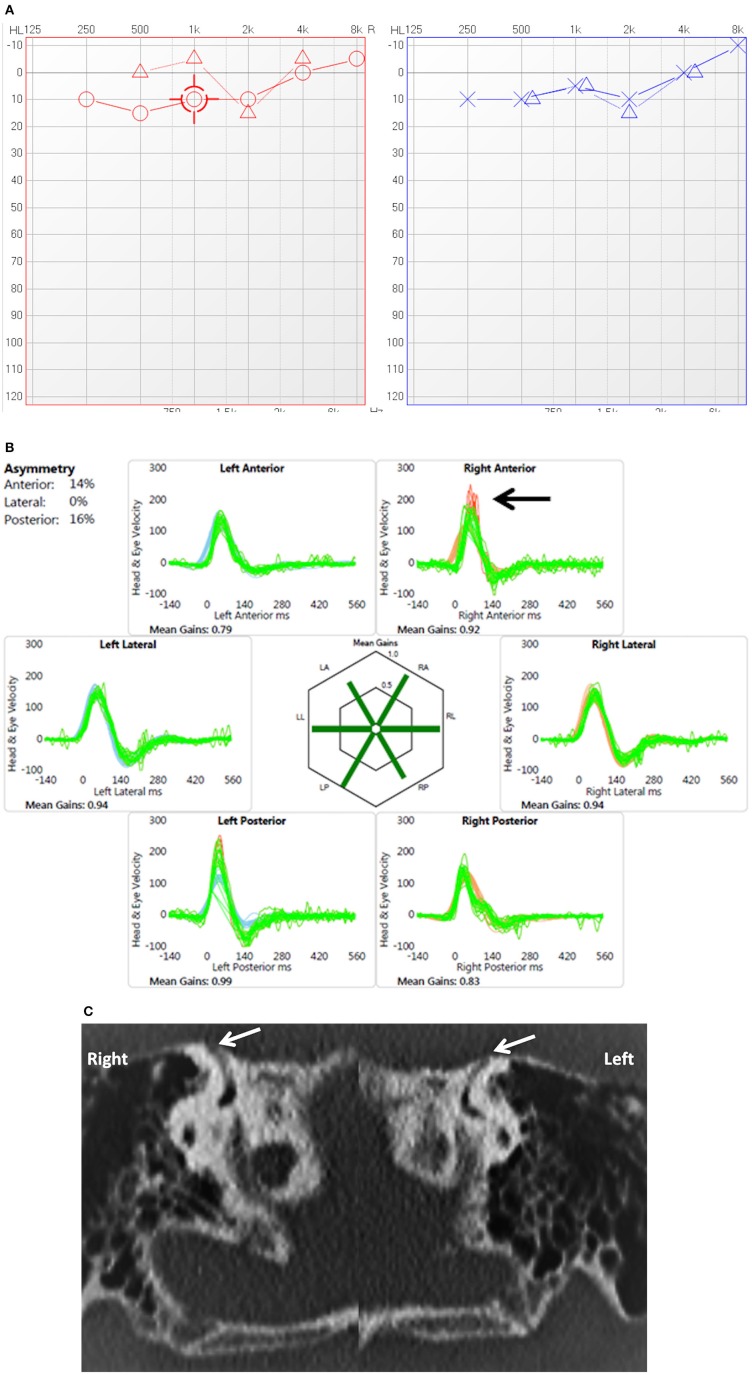
**(A)** PTA in child presenting with conductive dysacusis (child 7). **(B)** Video head impulse test in a child with covert saccades in the right superior semicircular canal (arrow). **(C)** Coronal reconstruction of High resolution CT scan shows dehiscence of the superior semicircular canal on both sides (arrows).

The observations for the full case series are given in Table 3. Figures 1–3 show representative cases.

**Table 3:** Clinical features and audiovestibular assessment in children with semicircular canal dehiscence; *n* = 13.

**Child**	**HS**	**BS**	**TWS**	**Tymp**	**SRT**	**OAE**	**VNG/VFT**	**VOR LLSC**	**VOR RLSC**	**VOR LASC**	**VOR RASC**	**VOR LPSC**	**VOR RPSC**	**AC/BC R**	**AC/BC L**	**Type HL**	**Saccade**	**Diagnosis**
1	Yes	Yes	Nil	Norm	Norm	Abn	Norm	1.16	1.09	0.55	0.75	0.85	0.69	55/46	58/46	Mixed	Yes	R PSCD
2	Nil	Yes	GET	Norm	Norm	Norm	Norm	0.67	0.48	0.62			1.46	7/−5	4/3	Nil	No	L SSCD R PSCD
3	Yes	Nil	Nil	Norm	Norm	Norm	Norm	0.96	0.96	0.41	0.85	0.82	0.51	27/7	10/na	CHL	Yes	R SSCD
4	Yes	Nil	Nil	Flat	Reduced	Abn	Norm	1.1	0.88					100/nt	17/4	SNHL	No	R SSCD
5	Yes	Yes	Nil	Norm	Norm	Norm	Abn	1.14	1.17	1.13	1.08	0.90	1.1	45/9	45/10	CHL	Yes	RSSCD
6	Nil	Nil	Nil	Norm	Norm	Norm	Norm	0.79	0.94	0.67	0.75	1.07	0.78	42/2.5	23/10	CHL	Yes	Bilateral SSCD
7	Nil	Nil	CD	Norm	Norm	Norm	Norm	0.94	0.94	0.79	0.92	0.99	0.83	9/6	7/6	Nil	Yes	Bilateral SSCD
8	Yes	Nil	Nil	Norm	Norm	Norm	Norm	0.86	0.83	0.50	0.89	0.82	0.39	25/14	45/20	CHL	Yes	R SSCD
9	Yes	Yes	Nil	Norm	Norm	Norm	Abn	0.86	0.90	0.73	0.48	0.54	0.47	36/30	41/26	Mixed	Yes	R SSCD R PSCD
10	Yes	Nil	Nil	Norm	Norm	Norm	Abn	0.82	0.90	0.73	0.88	0.75	0.73	17/15	21/15	Mixed	Yes	L SSCD
11	Nil	Yes	Auto/ tinnitus	Norm	Norm	Norm	Norm	0.95	0.90	0.41	0.37	0.66	0.59	12/7	17/9	Nil	Yes	R SSCD
12	Yes	Yes	CD	Norm	Norm	Norm	Norm	1.07	1.01	0.07	0.07	1.06	1.03	44/35	39/36	Mixed	Yes	Bilateral SSCD
13	Nil	Nil	Nil	Norm	Norm	Norm	Norm	1.18	0.99	0.65			0.98	4/10	55/51	SNHL	No	R SSCD

*Children in case group; Legends: HS, hearing symptoms; BS, balance symptoms; TWS, Third window symptoms; Tymp, tympanometry in dehisced ear/s; SRT, stapedial reflex test in dehisced ear/s; OAE, transient otoacoustic emission in dehisced ear/s; VNG/VFT, videonystagmography/other vestibular function tests; VOR, vestibulo-ocular reflex gain; LL/RL/LA/RA/LP/RP SC, left lateral, right lateral, left anterior, right anterior, left posterior, right posterior semi-circular canals; PTA AC R and L, pure tone audiometry air conduction thresholds averaged 500 Hz−4 kHz right and left; BC R and L, pure tone audiometry bone conduction thresholds averaged 500 Hz−4 kHz right and left; na, not performed; nt, no detectable threshold; HL, hearing loss; CHL, conductive hearing loss; SNHL, sensorineural hearing loss; GET, gaze evoked tinnitus; CD, conductive dysacusis; Auto, autophony; Tinn, tinnitus; Norm, normal; Abn, abnormal*.

Total number of children seen in the period of study were 580. Out of these, 13 children (2.2%) had radiological evidence of semicircular canal dehiscences. There were 6 boys and 7 girls in the case series. The average age in the female population was 11.28 (range 9 to 17 years) years whilst in the male population it was 9.83 years (range 6 to 14 years) in the case series presenting with semicircular canal dehiscences.

The 13 children with semicircular canal dehiscences involved either the superior semicircular canal (2.06% of the whole population) or the posterior semicircular canal (0.5% of the whole population). In 26 ears studied, SSCD was detected in 15 (57.69% of the semicircular dehiscence group) and PSCD was detected in 3. There were 4 children with bilateral semicircular canal dehiscence either both SSCD or with SSCD and PSCD (cases 2,6,7, and 12). One child presented with both superior and posterior canal dehiscence on one side (case 9). Ten (66.6%) out of the 15 SSCD were on the right as were all the 3 PSCD on the right.

Children presenting with symptoms of hearing loss, i.e., deficits in communication for example difficulties to hear or engage in conversation in the class room, difficulties to respond to orally delivered instructions were observed in 8; however, it must be noted that actual measured hearing losses (i.e., >20 dBHL in air conducted thresholds in any frequency) were detected in 10 as there was 1 child with unilateral sensorineural hearing loss who did not present with any symptoms of hearing loss (case 13) and 1 child with asymmetrical conductive hearing loss where the left ear showed better air conduction thresholds than the right did not complain of a hearing loss either (case 6). Measured hearing loss in terms of air conduction thresholds averaged between 500 Hz and 4 kHz therefore, was found in 76.9% of which 6 were bilateral and 4 were unilateral. Three children did not have a hearing loss. The hearing loss was mixed or conductive in 8 (80% of hearing losses and 61.5% of the whole case series) and was sensorineural in 2 children. These 2 children presented with SNHL on one ear (cases 4 and 13). The average air conduction hearing threshold between 500 Hz and 4kHz on the left ear was 29.4 dBHL and on the right was 32.5 dBHL (the mean of the summated averages of air conduction thresholds in each child) whilst the average bone conduction thresholds (the mean of the summated averages of bone conducted thresholds in each child) between 500 Hz and 4kHz was 20 dBHL on the left and 18 dBHL on the right.

As regards bone conduction thresholds, 5 children exhibited negative bone conduction thresholds in at least one frequency (cases 2,5,6,7, and 11) out of which 3 children (cases 2, 7, and 11) presented with third window symptoms as well and indeed fulfilled the criteria for an adult superior semicircular canal dehiscence syndrome (5). Two children (cases 5 and 6) had conductive hearing losses without any third window symptoms.

Tympanometry was normal in 12 children (92.3%) whilst stapedial reflexes were normal in 12 children as well. There was some otitis media with effusion in one child with unilateral profound sensorineural hearing loss (case 4) where tympanometry was flat with depressed stapedial reflexes on the dehisced side which was the side of the hearing loss. Transient otoacoustic emissions were normally recordable in 10 children on both ears (76.9%) and in 11 children on the dehisced sides (84.6%). They were absent in 2 children with sensorineural hearing loss on the ipsilateral ear of the hearing loss (cases 4 and 13) and in case 1 with a mixed hearing loss on both sides.

Three children were diagnosed with bilateral hearing losses but unilateral semicircular canal dehiscence (cases 1, 5 and 8), 2 children demonstrated bilateral semicircular canal dehiscences with bilateral hearing loss (cases 6 and 12). Four children (30.7%; cases 2,7,11 and 12) narrated classical third window symptoms. The hearing loss localized to the dehisced side in 11 ears but there were 5 ears where a hearing loss was detected with no radiographic evidence of dehiscence on the ipsilateral ear.

Six children presented with definite features of pediatric disequilibrium, i.e., symptoms as listed in Table 2 (46.15% of the study group and) while the rest 53.85% were asymptomatic. Three children had abnormal balance function tests not including the vHIT (head shake test, mastoid vibration test, head heave test, ocular counter rolling and subjective visual vertical test). Ten children (76.9%) had abnormal vHITs with either low VOR gain and saccades or with normal VOR gain and replicable or repeatable saccades (i.e., saccades that were consistent and multiple), 3 children had normal vHITs. The average gain of the left lateral semicircular canal in the case series was 0.96; the right lateral semi-circular canal was 0.92; the left superior semi-circular canal was 0.6; the right superior semi-circular canal was 0.70; the left posterior semi-circular canal was 0.85 and the right posterior semicircular canal was 0.8. Out of 18 ears with semicircular canal dehiscences, abnormal vHIT with saccades were detected in 12 (66.6% of all ears with semicircular canal dehiscence) and in 7 children they localized to the same canal in the affected side. It was observed that in 5 children, abnormal canal function was detected in other canals on the same side rather than the canal affected, i.e., for SCDS, the abnormal vHIT was seen in either the lateral or the posterior canals. In 2 children, there was unilateral vHIT abnormality in bilateral SCDS. All children had normal neurological and oculomotor function.

## Discussion

Although well-researched in the adult population, evidence relating to semicircular canal dehiscence in children is limited especially regarding clinical presentation and functional and objective vestibular quantification. The largest series to this date is by Meiklejohn et al. (13) who in their series including 19 children from the age of 2 months until 7 years observed a zero percent prevalence of the condition at the age beyond 3 years which is the starting age range of the current study. The majority of this case series presented with hearing loss and included variable comorbidities and cochleo-vestibular dysplasias as well. The current study in its methodology excluded children under the age of 5 years and children with comorbidities due to 2 main reasons—firstly, the dehiscence may be part of normal development under the age of 5 years (6, 15) and secondly cochleovestibular comorbidities might be responsible for the presenting phenotype rather than the dehiscence.

The prevalence of semicircular canal dehiscence in children over the age of 5 years is not resolved as yet especially given the variable observations in the limited evidence; it ranges from 0 to 13% (11, 13). In the current series, this was observed to be 2.2% overall with 2.06% in the superior and 0.5% in the posterior semicircular canals. Saxby et al. (21) observed a prevalence of 1.7% SSCD and a 1.2% PSCD in their series. This study was investigating primarily SNHL in their cohort. The current study differs from other studies as it includes children with both audiological and vestibular phenotypes and is the largest series of its kind. It can be noted that available evidence in the majority pertains to a radiological diagnosis rather than diagnosis based on third window features.

In the current series it was observed that the right sided superior semicircular canal was the most common canal that showed dehiscence (66.6%). Sanverdi et al. (22) in their series of 560 children with SNHL and varying degrees of otic capsule incomplete ossification observed a 7.5% ossification asymmetry. Asymmetry is a frequent attribute in all body organs although the small size in the current sample may be responsible for this observation. Bilateral dehiscences were detected in 4 children (30.7%). This has been reported sporadically in the literature (11, 16) and number only 3 in published literature. This observation augments previous evidence that indeed otic capsule dehiscence may be developmental due to a deficit in ossification of the bony otic capsule.

Measured hearing loss was observed in the current series in 76.9% of children of varying degrees. This appears to be one of the most common presenting features of semicircular canal dehiscence in children and the findings in the current study agreed with other published evidence(3, 11–13, 16, 17, 23). There were 3 children who had normal hearing thresholds. This can be deemed as an important finding as this has been hardly reported except in the series by Lee et al. (12) and Wenzel et al. (17), where one child in each case had normal hearing thresholds. Variability in presentation is a frequent observation in semicircular canal dehiscences in all age groups that is well-known. It is interesting to note that in the studied case series, these three children presented with symptoms of third window.

The main type of hearing loss in the current series was a mixed or conductive hearing loss in 80% of the children with hearing losses. This observation replicates those found in other studies for example Zhou et al. (3), Chen et al. (11), and Lee et al. (12). The reason for a mixed or conductive loss can be explained by the third window effect. Sensorineural hearing loss is not unknown either (16, 17, 24, 25) and in this series it was observed in 2 children. These children did not show any balance symptoms and the reason they were requested for a high resolution CT was because their MRI was normal as suggested in the aetiological diagnosis algorithm. The large series by Meiklejohn et al. (13) observed SNHL in the majority, however, they included cochleo-vestibular dysplasias in their series which were likely responsible for the hearing loss. It was also observed in the current series that hearing loss did not essentially correlate with radiographic evidence of a dehiscence; i.e., a deficit in hearing might not show a dehiscence on that side or vice versa. The authors believe that since the condition can evolve, a HRCT might show a frank dehiscence in future in these cases.

The issue of negative bone conduction threshold as a criteria for diagnosing SSCD (5) deserves special mention. This functional subjective test establishes a pathological third window effect. In our case series, only 5 children demonstrated negative bone thresholds, out of which, 3 complained of conductive dysacusis. Merchant et al. (26) in their cohort of 20 children observed that whilst bone conduction thresholds may be negative in superior semicircular canal dehiscence, it might not be true for all subjects presenting with the condition. Instead, they pointed out that a measured air bone gap in the presence of normal middle ear function was a more consistent finding that we have also observed in the current series including the 2 with negative bone conduction thresholds not presenting with conductive dysacusis.

All the 13 children in our study case series with sensorineural or mixed hearing losses were extensively investigated with MRI scans, genetic and blood tests that were unremarkable. Therefore, by the process of elimination, it was more likely than not that their hearing losses can be explained by the demonstration of a radiological semicircular canal dehiscence. This of course cannot be proven by an observational retrospective descriptive study like the current one, however, neither can it be said that a radiological dehiscence in this group can be incidental only.

Only 30% in our series described classical third window symptoms that are crucial for a diagnosis in an adult but seldom available from children. These include gaze evoked tinnitus or audible eye movements, conductive dysacusis, autophony, Hennebert's phenomenon or Tullio's phenomenon and pulsatile tinnitus. Many of these symptoms cannot be described by younger children, however, these symptoms if present will lead to some predictable behavior that can be picked up by parents. This observation raises an interesting point as apparent from the current descriptive study that a radiological diagnosis of semicircular canal dehiscence might not lead to a fully blown semicircular canal dehiscence syndrome in children that has been well-defined in adults. As a result, the diagnosis of a semicircular canal dehiscence in children can be guided by a holistic picture rather by obtaining third window symptoms.

Tympanometry was normal in the majority of our children that virtually eliminated any middle ear disorder with normal otoscopy in our case series. The finer nuances of tympanometry in semicircular canal dehiscences were investigated in the adult population by Castelucci et al. (27) who measured interaural differences but overall, the peak compliance average was within normal limits. The majority of their subjects also demonstrated normal stapedial reflexes as this current series also observed. The only child who presented with flat tympanometry and reduced reflexes had middle ear disease in addition to a profound SNHL on one side.

The publication by Thabet (28) investigating the finer nuances of transient OAE in semicircular canal dehiscences indicated that by and large OAE tend to be preserved in semicircular canal dehiscences. The authors feel that objectively establishing normal middle ear and cochlear function is very important to suggest and make a case for an inner ear problem explaining a conductive and a mixed hearing loss as is frequently found in semicircular canal dehiscences. In frank cases of SNHL, the pathology itself can lead to cochlear dysfunction over riding the third window effect generating absent OAEs as found in 2 children in the current series. The child with the mixed loss and absent OAE very likely developed a cochlear element to his hearing loss due to the same reason. No other cause was detected to account for the hearing loss in these children after intense aetiological investigations as given earlier.

46.15% of children in the current case series presented with features of disequilibrium which is a hallmark in the adult population, but again it must be remembered that obtaining a proper history is often surrogate and dependent on observational behavior. In other series by Chen et al. (11), Lee et al. (12), and Kanaan et al. (16), this had been reported and the likely reason is that in children cerebral plasticity leads to good compensation precluding symptoms even in cases of vestibular deficit. Nevertheless, if there is a history of compromised balance, the possibility of a third window must be raised.

VNG examination with and without optic fixation, rotatory chair tests and vestibulo-spinal tests as well as mastoid vibration tests were normal in the majority of children in the current case series (76.9%) suggesting that gravitational sensor and static, low and mid frequency angular motion sensor vestibular function tend to be preserved in semicircular canal dehiscence in children. To our knowledge, the present study is the largest study to date to employ these tests in semicircular canal dehiscence in the pediatric population.

The video head impulse test since first described by MacDougal et al. (29) has revolutionized the diagnosis of high frequency semicircular canal vestibular function. Whilst extensive research in the adult population has been carried out and new observations are evolving, its use in the pediatric population has not been studied widely and in this respect its use in the diagnosis of semicircular canal dehiscence in children has not been studied at all except in one isolated case report by Wenzel (17). Similarly in the adult population, the evidence is extremely limited. The current series utilized this test routinely on the premise that semicircular canal dehiscence in children may lead to high frequency vestibular involvement as a result of aberrant endolymphatic fluid movement generated by the third window. Carey et al. (30) assessed passive head thrust generated VOR gain with magnetic scleral coil search techniques in a group of patients with superior semicircular canal dehiscence in the preoperative and post-operative period. In the preoperative period, the VOR gain did not differ much from the normative data.

One important aspect to consider is the norms in the pediatric population due to the developing vestibular function. Wiener-Vacher and Wiener (31) published for the first time using the Synapsys system pediatric VOR gains in all 6 semicircular canals in the normal population and observed that although VOR gain increased as a function of age, their absolute numerical values are reasonably close to the adult population. However, a recent paper by Bachman et al. (19) suggested that whilst the VOR gain in the lateral semicircular canals match closely to those obtained in the adult population, the gain in the vertical canals can be distinctly lower in children. Bachman et al. (19) used the ICS Impulse system which is fundamentally different form the Synapsys system. This study utilized the ICS Impulse system and observed similar VOR gains as the Bachman study in a normal cohort (not a part of this audit). Saccades were considered as the most important indicator of vestibular dysfunction as Perez- Fernandez and Eva-Nunez (32) and Korsager et al. (18) had shown that saccades can be observed with normal VOR gain values during compensation that suggests persistent VOR deficits. Another important consideration is whether these saccades are artifactual or not which is a common problem to be encountered especially in the vertical canals (33). All the vHITs in the current study were analyzed and reviewed by the authors who are experienced clinicians and had performed over 800 vHITs in the pediatric population. They believe that just like an auditory brainstem response, vHITs need to be interpreted on an individual basis and correlated with the overall phenotype of the condition being investigated.

Replicable and repeatable saccades (consistently occurring multiple saccades up to 400 ms) were observed in 10 children (76.9%) whilst in 3 there were no discernible saccades. These saccades localized fairly accurately to the sides and the dehisced canals but with some exceptions. In 1 child with bilateral dehiscence, saccades localized to the lateral canal of one side only. In 1 child, they were observed in the left lateral semicircular canal for a left sided SSCD and in another child, in the right lateral semicircular canal for a right sided SSCD. Furthermore, in 2 children, they were observed in the right posterior semicircular canal for right SSCDs. In 1 child, the dehiscence was on the opposite side of an SNHL who had normal vHIT. Again, these outlying observations may suggest the variability of presentation of the condition. The saccades detected from vHIT in non-dehisced canals may be due to secondary effects generated by an aberrant endolymphatic fluid dynamics in response to the third window affecting the VOR. It is not known whether in the pediatric population, there could be a continuous damping effect of the VOR. Therefore, from this study, it appears that saccades may be important to assess high frequency vestibular function in semicircular canal dehiscences in children. This has not been reported before and indeed the authors feel that vHIT could be incorporated in the diagnostic process to take a decision whether to request HRCT for confirmation of diagnosis of a third window along with other clinical features.

The current series observed that measured hearing loss which is a functional measure and abnormalities in the video head impulse test which is an objective measure were present in a high percentage of children with semicircular canal dehiscences (76.9%). The majority of children with measured hearing losses complained of a functional deficit in hearing. However, only about 46.15% presented with functional deficits in balance in spite of an abnormal objective measurement. These observations suggest that semicircular dehiscences in children may tend to possess more functional hearing deficits than functional disequilibrium and the hearing loss may correlate to a measured objective balance deficit rather than a functional balance deficit.

The children in the current series did not undergo VEMPs to diagnose semicircular canal dehiscences as these tests were not available initially but were procured toward the end of the study period. It has been shown that otolith function may be enhanced in semicircular canal dehiscence (34) and measured by VEMPs. This remains an important tool for diagnosis of semicircular canal dehiscences to objectively confirm the third window effect in the adult population. However, in children, the evidence is still emerging. One of the difficulties in children is non-cooperation and achieving good muscle contraction for a robust response. As yet standardized norms derived from a sizeable population have not been published. It is likely that latencies and amplitudes will be less than in the adult population (35). Zhou et al. (36) studied VEMPS in children and observed that in inner ear structural abnormalities (for example enlarged vestibular aqueduct and semicircular canal dehiscences), VEMPs have lower thresholds and increased amplitude as one would expect in adults. However, it is not clear from this study as to what is defined by a structural inner ear abnormality. A structural inner ear abnormality is a vast entity and the authors did not mention as to how many of these actually had a third window.

VEMPs confirm the presence of a third window effect in the adult population that has been established (5). Ward et al. (5) in 2017 proposed the diagnostic criteria for third windows in adults that include high resolution CT confirmation and at least one third window feature enumerated previously and at least one of negative bone conduction thresholds in pure tone audiometry, enhanced VEMP responses and elevated summating potential to action potential ratio in electrocochleography in the absence of sensorineural hearing loss. Therefore, it can be noted that VEMPs in adults is one of the diagnostic criteria and not an obligatory one, although the paper mentioned that the authors believed that VEMPs are essential for diagnosis. In any case, VEMPs to demonstrate a third window effect in children may be required especially since children may not present with third window symptoms as discussed later.

Three children in the current case series presented with all 3 criteria—a negative bone conduction threshold, third window symptoms and a radiological evidence of semicircular canal dehiscences. Two children with conductive hearing losses and radiological evidence of SSCD also demonstrated negative bone conduction thresholds. The majority of children with hearing losses do not fulfill the adult criteria nor do they present with third window symptoms, yet they demonstrate radiographic evidence of a dehiscence. As discussed earlier, a cochlear hearing loss can be a feature of semicircular dehiscences in children and this hearing loss can serve as a phenotype for the condition. The extensive study by Lagman et al. (37) analyzing data in existing literature pertaining to SSCD in children concluded that hearing loss was the commonest indication for performing a high resolution CT scan that showed SSCD; however, it must be noted that about 25% children in this analysis had other causes explaining the hearing loss. The current series is the first to exclude this comorbidity group by performing extensive investigation for hearing loss and also employed other features for example third window symptoms and disequilibrium and vestibular function tests as indicators for further imaging. These observations suggest that a radiologically demonstrated canal dehiscence may not result in a fully overt dehiscence syndrome in children.

The important difference between adults and children is the lack of third window symptoms that suggests that the pathophysiology of a semicircular canal dehiscence (not to be confused with a superior semicircular canal dehiscence syndrome) may differ from that in an adult. The reason for this could be attributed to the observation that endolymphatic movement in the child's ear and its response to the third window can be different as compared to an adult, a suggestion that can be gleaned from minor traumatic brain injuries in children (38). Therefore, the real effects of a third window in a pediatric population remain to be established. This in turn might influence VEMP findings in children with the condition.

As indicated earlier that high resolution CT may over diagnose the condition especially in children as normal otic capsule ossification follows a chronological pattern. Therefore, to decide whether a radiological diagnosis is indeed the cause for the clinical features that a child presents with is a matter of fine judgment and expertise. Thus, diagnosis of semicircular canal dehiscence in children must be guided by the whole clinical picture rather than by one investigation alone.

The results in this audit observed that a significant proportion of children with apparent radiological diagnosis of semicircular canal dehiscence may not fulfill all the diagnostic criteria for the condition as proposed in adults with the algorithm employed in the study. This is due to the absence of typical third window features. The CT scan findings can be incidental and not related to the phenotype. Hence it may be necessary to establish a third window effect if possible by objective means in children. This can be achieved by VEMPS and thus this audit leads to the recommendation that to investigate semicircular canal dehiscences in children further, other tests can add to the ones performed in the study to obtain a better idea about the condition in children. VEMPs in spite of its limitations in the pediatric population need to be explored to demonstrate this pathological third window effect in children who otherwise present without any third window features about which there is limited evidence. Furthermore, functional vestibular tests for example the functional head impulse test (39) and the gaze stabilization test (40) may be attempted to correlate a functional phenotype that is yet to be investigated in children. Demonstration of a third window effect has implications regarding operative intervention that is well-established in adults. In our experience, surgery is seldom required in children and they respond well to audiovestibular rehabilitation with amplification, customized vestibular rehabilitation and with cognitive behavioral therapy when needed.

There is a possibility that a dehiscence picked up in childhood may lead to frank third window features in the future, there is no way of predicting this in the absence of adequate evidence. Again, this could be due to bony remodeling. Consequently, these children and parents can be counseled to deal with emerging problems in the future. This underpins the holistic approach to pediatric medicine, it is not just invasive management or medical management but preparing the child from any eventuality in the wider sense. A robust transition plan will incorporate this information to inform adult services when the child graduates from pediatric to adult services.

The weaknesses of this study include a small sample size given the rarity of semicircular canal dehiscence in children and that this was a retrospective audit in the first instance. However, since the study group was assessed by the authors only, there was continuity of care thus eliminating one of the important biases of a retrospective study. There was consistency in the process and influence by confounding logistic variables were negligible as the analysis looked into a defined anatomical abnormality which is independent of any modification by these variables. This study did not pose any hypothetical research question and did not attempt to confirm statistically valid observations. This study presents a series of observations that became apparent on a retrospective case note review and draws conclusions that might lead to further research in diagnosing a rare pathology in children.

## Conclusions

The pathological entity of semicircular canal dehsicences in children remains rare. High index of clinical suspicion to demonstrate the condition by dedicated imaging is suggested by good anamnesis, the presence of a conductive or a mixed hearing loss in the presence of normal middle ear and cochlear function and abnormal vestibular function tests. Sensorineural hearing loss may be a presenting feature. It observes that diagnosis of semicircular canal dehiscences in children depended on a number of functional and objective parameters. It does not suggest that any one test is best for diagnosis but rather provides an overview from a retrospective case note analysis that the phenotype in children is variable and diagnosis is a matter of clinical judgment. Furthermore, this study observes that an anatomical semicircular canal dehiscence might not present with a frank semicircular canal dehiscence syndrome characterized by the constellation of symptoms well-described in adults.

## Data Availability

All datasets generated for this study are included in the manuscript and/or the supplementary files.

## Author Contributions

SD and SR were the responsible clinicians for the children included in the case series. SD collated the data. SD and SR collaborated to write the manuscript and peer reviewed clinical findings and the manuscript drafts.

### Conflict of Interest Statement

The authors declare that the research was conducted in the absence of any commercial or financial relationships that could be construed as a potential conflict of interest.
